# High-Throughput Microscopy Analysis of Mitochondrial Membrane Potential in 2D and 3D Models

**DOI:** 10.3390/cells12071089

**Published:** 2023-04-05

**Authors:** Caterina Vianello, Federica Dal Bello, Sang Hun Shin, Sara Schiavon, Camilla Bean, Ana Paula Magalhães Rebelo, Tomáš Knedlík, Emad Norouzi Esfahani, Veronica Costiniti, Rodrigo S. Lacruz, Giuseppina Covello, Fabio Munari, Tommaso Scolaro, Antonella Viola, Elena Rampazzo, Luca Persano, Sara Zumerle, Luca Scorrano, Alessio Gianelle, Marta Giacomello

**Affiliations:** 1 Department of Biology, University of Padova, Via Ugo Bassi 58B, 35131 Padova, Italy; 2 Department of Medicine, University of Udine, Piazzale Kolbe, 33100 Udine, Italy; 3 Department of Molecular Pathobiology, New York University College of Dentistry, New York, NY 10010, USA; 4 Department of Biomedical Sciences, Via Ugo Bassi 58B, 35131 Padova, Italy; 5 Pediatric Research Institute, Città della Speranza Foundation, Corso Stati Uniti 4 F, 35127 Padova, Italy; 6 Laboratory of Tumor Inflammation and Angiogenesis, Center for Cancer Biology, Department of Oncology, KU Leuven, B3000 Leuven, Belgium; 7 Oncohematology, Department of Women’s and Children’s Health, University of Padova, Via Giustiniani 3, 35128 Padova, Italy; 8 Department of Medicine, University of Padova, Via Giustiniani 2, 35128 Padova, Italy; 9 Veneto Institute of Molecular Medicine, Via Giuseppe Orus 2, 35128 Padova, Italy; 10 National Institute for Nuclear Physics, Padova Division, Via Marzolo 8, 35131 Padova, Italy

**Keywords:** TMRM, mitochondrial membrane potential, spheroids, co-culture, NSCs, single muscle fibers, machine learning

## Abstract

Recent proteomic, metabolomic, and transcriptomic studies have highlighted a connection between changes in mitochondria physiology and cellular pathophysiological mechanisms. Secondary assays to assess the function of these organelles appear fundamental to validate these -omics findings. Although mitochondrial membrane potential is widely recognized as an indicator of mitochondrial activity, high-content imaging-based approaches coupled to multiparametric to measure it have not been established yet. In this paper, we describe a methodology for the unbiased high-throughput quantification of mitochondrial membrane potential in vitro, which is suitable for 2D to 3D models. We successfully used our method to analyze mitochondrial membrane potential in monolayers of human fibroblasts, neural stem cells, spheroids, and isolated muscle fibers. Moreover, by combining automated image analysis and machine learning, we were able to discriminate melanoma cells from macrophages in co-culture and to analyze the subpopulations separately. Our data demonstrated that our method is a widely applicable strategy for large-scale profiling of mitochondrial activity.

## 1. Introduction

Mitochondria are key cellular organelles, characterized by a high level of complexity in both their structure and function. They are surrounded by a highly dynamic double-membrane system (including the outer and inner mitochondrial membrane, OMM and IMM, respectively), the plasticity of which is promoted by continuous morphological changes due to the fission and fusion processes [1]. The shape of the mitochondrial network ranges from long interconnected tubules to fragmented organelles, depending on the cell energetic needs and exogenous/endogenous stimuli [1]. Mitochondrial reshaping controls different cellular functions, such as respiration, generation of ATP and reactive oxygen species (ROS), Ca^2+^ homeostasis, and adaptation to various stress conditions [1]. The generation of ATP uses the difference in the transmembrane electrochemical proton potential, which is derived from the combination of proton chemical gradient (ΔpH) and membrane potential (ΔΨm) generated by proton pumps. ΔΨm is commonly assumed to be a readout of mitochondrial well-being and function [2]. Many factors can alter ΔΨm and thereby lead to defects in the maintenance of mitochondrial homeostasis, which would ultimately be reflected in changes in the overall cell physiology [2,3]. For example, the entry of cations (primarily Ca^2+^) or solutes into mitochondria, as well as changes in the IMM lipid composition and mitochondrial ultrastructure, can result in the collapse of ΔΨm, thereby hampering ATP synthesis and ultimately participating in the onset of human disorders [4,5,6,7]. Thus, a method to measure basal ΔΨm reliably in live samples, in cells exposed to stress/stimuli, or in pathological contexts is fundamental to investigating cellular and organism pathological and physiological features. Currently, several fluorescent probes are available to monitor ΔΨm [8]: DiOC6(3), rhodamine 123 (Rh123), JC1, 2-(4-(dimethylamino)styryl)-1-methylpyridinium iodide (DASPMI [9]), tetramethylrhodamine ethyl (TMRE), or methyl (TMRM) esters. They all accumulate into the mitochondrial matrix in a manner proportional to ΔΨm, according to the Nernst equation [10], and their redistribution from inside/out or outside/in is consistent with the changes in ΔΨm.

TMRE/TMRM are considered the most reliable probes because they are less prone to artifacts associated with mitochondrial membrane binding or inhibition of the electron transport chain [8,11]. Since its discovery, TMRM has been routinely used to measure ΔΨm kinetics in single cells with conventional fluorescence microscopes. Recently, two-photon microscopy has allowed the use of TMRM in in vivo studies [12]. Furthermore, TMRM-based super-resolution microscopy has confirmed the existence of a broad ΔΨm heterogeneity within the same mitochondrion, as previously postulated by using DASPMI and JC1 dyes [13,14,15,16].

TMRM can be used in two modes: quenching and non-quenching modes. In the quenching mode, cells are loaded with a large concentration of the dye, which accumulates in the mitochondrial matrix as a factor of the ΔΨm. The aggregation of large amounts of TMRM molecules in the matrix causes quenching of its fluorescence [8]. Mitochondria depolarization leads to increased leakage of the dye from the organelles, thereby leading to an increase in the cytoplasmic fluorescence signal. The opposite holds true in the case of TMRM non-quenching measurements: at lower TMRM concentrations (5–20 nM), the dye accumulates in the mitochondrial matrix, and the mitochondrial signal decreases in the event of mitochondrial depolarization. Importantly, the accumulation of organic dyes into subcellular compartments can be influenced by their partially hydrophobic nature, which causes their non-specific binding to phospholipids and fluorescence artifacts [8,17]. Moreover, as quenching is not a linear event, it can also be used to detect wide ΔΨm changes, while more subtle and real-time changes in ΔΨm can be reliably detected only via non-quenching experiments [8]. To elicit changes in ΔΨm, two compounds are commonly used: oligomycin and FCCP (carbonyl cyanide 4-(trifluoromethoxy)phenylhydrazone). Oligomycin is an inhibitor of ATP synthase; thus, oligomycin induces mitochondrial hyperpolarization by blocking proton re-entry. Thus, changes in ΔΨm to oligomycin are lower in mitochondria with impaired respiration or with a “proton-leaky” membrane. On the other hand, the protonophore FCCP depolarizes the ΔΨm, thereby disrupting ATP synthesis by transporting protons across the mitochondrial inner membrane. Multiplexed high-content analysis of some mitochondrial parameters was proposed a few years ago [18,19]. However, the proposed approach includes only a single time point of ΔΨm measurement in cell monolayers. Measurements of ΔΨm kinetics in complex biological samples, such as mixed cultures of different cell populations or of primary cells, or 3D biological models such as spheroids or isolated muscle fibers, have been so far hampered by the time-consuming nature of sample preparation and of time-lapse imaging in conventional microscopy. Indeed, it takes more than one hour per sample to obtain kinetic ΔΨm measurements, limiting the number of samples analyzed per day. This leads to greater variability in datasets hampering the comparison of different experimental conditions.

Therefore, we set out to develop an unbiased approach based on high-content microscopy suitable for both 2D and 3D models. This novel high-throughput quantification not only overcomes the above-mentioned issues but also offers the possibility of coupling ΔΨm kinetics measurements with quantitation of other subcellular parameters.

## 2. Materials and Methods

### 2.1. Cell Culture

#### 2.1.1. Human Cell Lines

Human dermal fibroblasts PCS-201-012™ (HDFa) and epithelial melanoma A375 cells were cultured in Dulbecco’s modified Eagle’s (DMEM) medium (Life Technologies #12800-082), supplemented with 10% fetal bovine serum (FBS), 100 μM penicillin/streptomycin (Pen/Strep), and 1% L-glutamine and non-essential amino acids (Gibco, #11140-035). Lung fibroblasts IMR-90 were cultured in MEM α medium with GlutaMAX^TM^ (Life technologies #32561-029), supplemented with 10% FBS, Pen/Strep, and 1% L-glutamine and non-essential amino acids (Gibco, #11140-035). Cells were grown in 25 cm^2^ flasks in a humidified incubator at 5% CO_2_ at 37 °C. For sub-culturing, after being washed with PBS, cells were detached with 1 mL 0.05%/0.02% trypsin/EDTA before being resuspended in complete media, and cells were plated at the desired concentration.

#### 2.1.2. Primary Cell Lines

##### Isolation and Differentiation of Human Macrophages

Human monocytes were isolated from buffy coats obtained from healthy blood donors (anonymously provided by the Transfusion Centre of the University Hospital of Padova), after written informed consent from each donor for the use of surplus blood products for research purposes. In detail, blood was first diluted 1:4 with sterile PBS without Ca^2+^ and Mg^2+^ (Lonza). Then, 5% dextran (Sigma-Aldrich) was added diluted at a ratio of 1:5, and erythrocytes were sedimented for 30 min. The supernatant containing white blood cells was collected and centrifuged for 15 min at 50× *g*. The cell pellet was resuspended in 15 mL of sterile PBS, stratified on Ficoll-Paque (GE Healthcare), and centrifuged for 30 min at 400× *g* without brake. Cells were recovered and washed for 15 min at 311× *g* and then stratified on Percoll (GE-Healthcare) gradient (15.76 mL RPMI 1640, 10% FCS (*v*/*v*), HEPES 4 mM (Lonza), Pen/Strep, 285 mOsm; 15.54 mL 10% Percoll in 10× sterile PBS 285 mOsm), and centrifuged 30 min at 400× *g* without the brake and accelerator. Monocytes were recovered and washed for 15 min at 311× *g,* and then, they were resuspended in RPMI 1640, 2% FBS, Pen/Strep. Afterwards, the cells were seeded at 10 × 10^6^ density in tissue culture dishes and separated from the contaminating lymphocytes via adherence (1 h at 37 °C). Adherent monocytes were extensively washed with a medium to remove residual non-adherent cells. After washing, cells were incubated in RPMI 1640, 20% FBS, HEPES 4 mM, Pen/Strep, and 100 ng/mL M-CSF (Miltenyi Biotec) for three days. After 3 days, half of the medium was replaced with fresh medium supplemented with 100 ng/mL M-CSF.

#### 2.1.3. 3D Models

##### LUHMES Cells and Formation of Spheroids

For proliferation, LUHMES cells were kept in flasks coated with poly-L-ornithine (0.1 mg/mL; 4 °C, overnight; Sigma-Aldrich) in a DMEM/F12 medium (Sigma-Aldrich) with 1% N2-supplement (Thermo Fisher Scientific, Cat#: 17502048), 2 mM L-Glutamine (Thermo Fisher Scientific), and a 0.04 μg/mL human basic fibroblast growth factor (bFGF, Gibco, Cat#: 13256-029). For differentiation, the cells were seeded in a differentiation medium at a density of 100,000 cells per cm^2^ in the cell culture dishes coated with poly-L-ornithine (50 μg/mL; 4 °C, overnight; Sigma-Aldrich), followed by coating with human fibronectin (1 μg/mL, 37 °C, overnight; Sigma-Aldrich). The differentiation medium consisted of a DMEM/F12 medium with 1% N2-supplement, 1 μg/mL tetracycline (Sigma-Aldrich), 1 mM dibutyryl cAMP (Sigma-Aldrich), and a 2 ng/mL glial cell-derived neurotrophic factor (GDNF) (Sigma-Aldrich, Cat#: G1777). To handle LUHMES spheroid culture, between 1000 and 3000 cells were seeded into ultra-low attachment, U-shaped, 96-well plate (CellCarrier Spheroid ULA 96-well Microplates, Perkin Elmer, Cat#: 6055330) with 100 μL of LUHMES proliferating medium.

##### Myofibers

Single myofibers were isolated via enzymatic dissociation from tibialis anterior (TA) and extensor digitorum longus (EDL) mouse muscles of C57BL/6 mice, as previously described [20]. All procedures were authorized by the Italian Ministry of Health (approved protocol: 383-2015-PR to L.S.). Briefly, muscles were dissociated 45 min at 37 °C in 1% (*w*/*v*) type I collagenase (Sigma-Aldrich). Then, the dissociated muscles were transferred in DMEM (low glucose; Invitrogen, Waltham, MA, USA) on a horse serum (Invitrogen)-coated 10-cm dish (Falcon; BD Biosciences) and gently triturated with a wide-bore pipette to release single myofibers. Myofibers were individually collected under a stereo microscope.

### 2.2. TMRM Fluorescence Measurements Using High-Content Microscopy

For analysis of ΔΨm, cells were plated into 384- or 96-well plates (CellCarrier, PerkinElmer). After 24–48 h in culture, cells were rinsed with 10 mM HEPES buffered saline solution (HBSS—Hank’s Buffered Salt Saline, *Invitrogen*, pH 7.4 with 1 g/L of glucose) and subsequently incubated with 20 nM TMRM (Thermo-Fisher Scientific) in the presence of 1 μM cyclosporine H (Sigma) for 30 min at 37 °C; the same buffer was used during image acquisition. Alternate brightfield, Hoechst 33,342 (Ex:360–400/Em:410–480 nm), and TMRM fluorescence (Ex:520–550/Em:560–630 nm) images were acquired sequentially, by using different magnification air objective (10 × −20 × −40 ×) of the high-content screening (HCS) imaging system Operetta^®^ and Harmony^®^ 4.8 analysis software (PerkinElmer). After acquisition of basal fluorescence intensity, cells were treated with oligomycin and then with FCCP. The analysis was performed by means of Harmony^®^ software (PerkinElmer), as summarized in Supplementary Figures S1, S2, S4, and S5. Briefly, image segmentation was performed via detection of regions of interest (ROI; each ROI corresponds to a single cell) in the Hoechst 33,342 channel. Background-corrected TMRM fluorescence intensity was then measured per each ROI and averaged. In the next paragraphs, details for each model will be provided.

#### 2.2.1. TMRM Fluorescence Measurement in Human Fibroblasts

In total, 3000 cells per well were seeded on 384-well plates (CellCarrier, PerkinElmer). Image acquisition was performed with a 40× objective. Image segmentation was performed as described in detail in Supplementary Figure S1. More than 100 cells/biological replicates were analyzed: 5 biological replicates for HDFa and 6 biological replicates for IMR-90.

#### 2.2.2. TMRM Fluorescence Measurement in LUHMES

In total, 3000 cells per well were seeded on 96-well plates (CellCarrier, PerkinElmer). Image acquisition was performed with a 10× objective, with Z-stack, acquiring 5 different planes at 13 μm distance. Image segmentation was performed as described in detail in Supplementary Figures S2 and S3. More than 300 objects per class of single cells and grouped cells and more than 40 aggregates per biological replicate were analyzed; biological replicate = 3. For spheroids, at least 10 objects within 3 biological replicas were analyzed.

#### 2.2.3. TMRM Fluorescence Measurement in Myofibers

Isolated myofibers from wild-type C57BL/6 mice were plated in 384-well plates (CellCarrier, PerkinElmer). Z-stack image acquisition was performed with a 10× objective long working distance, step size 45 μm (Operetta, Perkin Elmer). After basic flatfield correction, brightfield images were processed via a sliding parabola (curvature 3, see Supplementary Figure S4). Three-dimensional reconstruction from the respective Z-stacks (45 μm step size) was used to define fiber-positive image areas and subsequent identification of true fibers based on morphology and texture properties (Supplementary Figure S4). Background-corrected TMRM fluorescence intensity was obtained by subtracting the average TMRM intensity of background ring regions from the TMRM of each individual fiber. Number of objects analyzed = more than 5 per independent replicate; number of replicates = 5–6.

#### 2.2.4. TMRM Fluorescence Measurement in Cell Co-Cultures

Cancer cells were seeded as 1000 cells/well. After 24 h of polarization, macrophages were harvested and seeded in 96-well optical plates (Eppendorf, cat# 0030741030) at a concentration of 4000 cells/well. TMRM data were acquired with a 20× objective. In co-cultures, Hoechst staining properties were exploited for image segmentation (Supplementary Figure S5). Images were segmented based on Hoechst staining (see also Supplementary Figure S5). Several morphological, intensity, and texture parameters were quantified in the Hoechst channel per each individual object and were exploited by means of the machine-learning approach to correctly distinguish macrophages from cancer cells. After classification, with Harmony^®^ Imaging and Analysis software, the fluorescence of TMRM for each recognized cell was obtained, and its average intensity was calculated at each timepoint and normalized to the basal value of control cells.

### 2.3. Machine Learning Approaches in Co-Cultures of Cancer Cells-Macrophages

The machine learning algorithm used was a Deep Neural Network (DNN)-built alternating Dropout layer after each Dense layer, starting with a Batch Normalization layer. The same model was used for all the combinations between population A and B. The DNN networks were implemented by using the Keras framework [21] with the TensorFlow back-end [22]. The hyper-parameters of the network (e.g., number of nodes per layer, dropout rate, normalization moment, kernel initialization, and optimizer) were tuned to reach best accuracy in a blind test dataset. The final chosen architecture consisted of five couples of dense layers plus a dropout layer; the number of nodes for the dense layers were 350, 224, 128, 96, and 96; and the dropout rate was 0.01 for all the layers. The optimization was performed by using a stochastic gradient descent (SDG) optimizer with a learning rate of 0.01. ReLu activation was used for the hidden layer, while a sigmoid was used for the output node. The model was trained for a maximum of 250 epochs; early stopping with a patience parameter of 25 epochs on the loss in the test set was used. To reduce the number of used features, we tested the approaches with a set of features selected by obvious biological significance (Supplementary Datasets S1–S4) or by means of the Permutation Importance algorithm of the eli5 Python library (which selects the most important features by measuring how the objects score decreases when a feature is not available: https://eli5.readthedocs.io/en/latest/blackbox/permutation_importance.html (accessed on 1 April 2020)). The final analysis shown in Supplementary Figure S5 takes into account all the available features. The corresponding Python scripts are available here as Supplementary Datasets and include Supplementary Dataset S1 (DNN_Classification.py: the major script, which is necessary for the training and generation of the DNN); Supplementary Dataset S2 (Utilities.py: a set of functions to complement the main script); Supplementary Dataset S3 (confM1.py, this dataset contains an example of data organization and configuration for one population of macrophages); Supplementary Dataset S4 (DNN_Eval.py, a script to generate output evaluations). Each cell was assigned with a score (ranging from 0 to 1), which indicates the probability of belonging to a specific cell population (e.g., cancer cells or macrophages); the higher the score, the higher the probability of belonging to the target population. The results were filtered based on the score assigned to each individual cell, and all the objects classified as “macrophages” or “cancer cells” with a score lower than 0.6 were discarded from our statistical analyses.

### 2.4. Cell Cycle Analysis of Cancer Cells

To perform the analysis of cell cycle phases, we took advantage of Hoechst nuclear staining. To calculate the percentages of cells in different cell cycle phases, we measured the relative frequency distribution of Hoechst intensity per cell (sum of nuclei intensity per well) via GraphPad software (GraphPad Software, San Diego, CA, USA).

## 3. Results

### 3.1. Measurements of ΔΨm Kinetics Based on High-Content Microscopy

**Figure 1:**
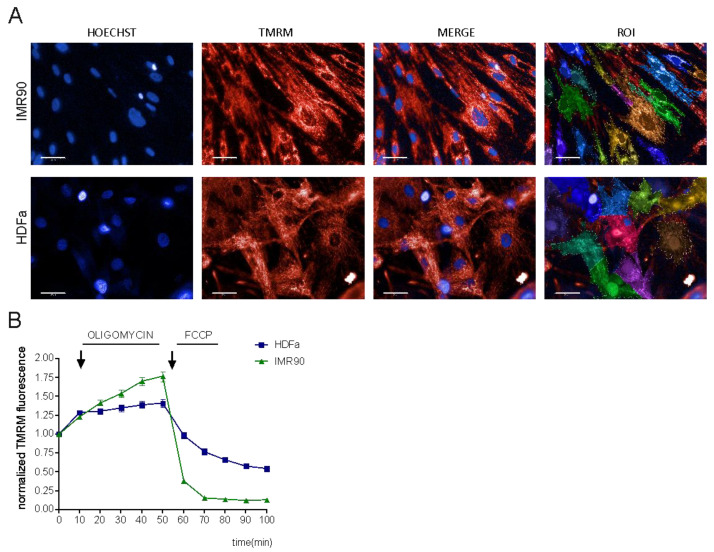
High-content measurement of ΔΨm in cell cultures. (**A**) Representative images of primary human fibroblasts (HDFa, IMR-90) stained with TMRM and Hoechst (scale bar 50 µm). (**B**) Mitochondrial membrane potential (ΔΨm) kinetics of human fibroblasts. To study the changes in ΔΨm, the cells were treated with oligomycin and FCCP. Data represent the mean ± SEM of 5–6 biological replicates.

As mentioned before, TMRM is the most reliable ΔΨm probe to date. We aimed to standardize a high-content image analysis methodology to measure ΔΨm; therefore, we decided to analyze ΔΨm kinetics via a TMRM non-quenching approach. Initially, we set up the procedure in 2D models, e.g., cell monolayers such as dermal fibroblasts (HDFa, obtained from ATCC) and lung fibroblasts (IMR-90) (Figure 1A). First, we incubated the cells with 20 nM TMRM and 25 μg/mL Hoechst 33,342 and performed high-content imaging by means of the automated fluorescence microscope (Operetta^®^, Perkin Elmer). Fully automated measurement of TMRM fluorescence intensity was performed at different timepoints: first in basal conditions and then upon exposure to oligomycin and FCCP. As mentioned previously, oligomycin (inhibitor of ATP synthase) induces mitochondrial hyperpolarization, while FCCP (uncoupler of mitochondrial oxidative phosphorylation) causes ΔΨm collapse and decrease in the fluorescence intensity (Figure 1B). High-content imaging of ΔΨm was followed by fully automated image analysis (Harmony 4.8 software, Perkin Elmer). The workflow of the analysis includes sequential steps (Supplementary Figure S1): at first, Hoechst images were segmented for optimal detection of individual nuclei, thereby providing the possibility to detect ΔΨm at both single-cell and, upon clustering, population levels (Supplementary Figure S1). TMRM fluorescence intensity was measured both within the cells and in the background ring region surrounding each cell: the background-corrected TMRM intensity was used as a readout of ΔΨm changes in each object of interest. With this approach, we were able to follow the kinetics of ΔΨm in dermal and lung fibroblasts: we could observe the hyperpolarization induced by oligomycin and depolarization upon exposure to FCCP in both cell lines, but with a faster ΔΨm decrease in IMR-90 cells (Figure 1B).

### 3.2. ΔΨm Analysis in the Neural Precursor Cells and Spheroids

A compelling challenge in high-content microscopy is to perform live imaging and automated quantification of intracellular parameters in 3D models. In the present study, we propose an image analysis workflow that allows the analysis of both 2D and 3D cell models at once upon image segmentation based on simple morphological and texture parameters. To achieve this aim, we took advantage of the neural precursor Lund Human Mesencephalic (LUHMES) cell line, which has been previously used to generate spheroids [23]. Spheroids, as well as organoids, are often characterized by structural variability even if grown in parallel with the same cell population: this was also the case for LUHMES, which yielded different types of 3D structures even within the same well. We wondered whether LUHMES physiology could change depending on the extent of their inter-cellular interactions. To address this point, we categorized the diverse cell structures into classes, which were defined upon image segmentation based on Hoechst nuclear staining (see the Materials and Methods section as well as Supplementary Figure S2). Our image analysis workflow allowed us to distinguish four populations, depending on the area of the cell cluster: single cells, grouped cells, big aggregates, and spheroids. Automated image analysis followed by the quantification of background-corrected TMRM fluorescence intensity revealed that mitochondria membrane potential differs based on the size of the cell clusters.

**Figure 2:**
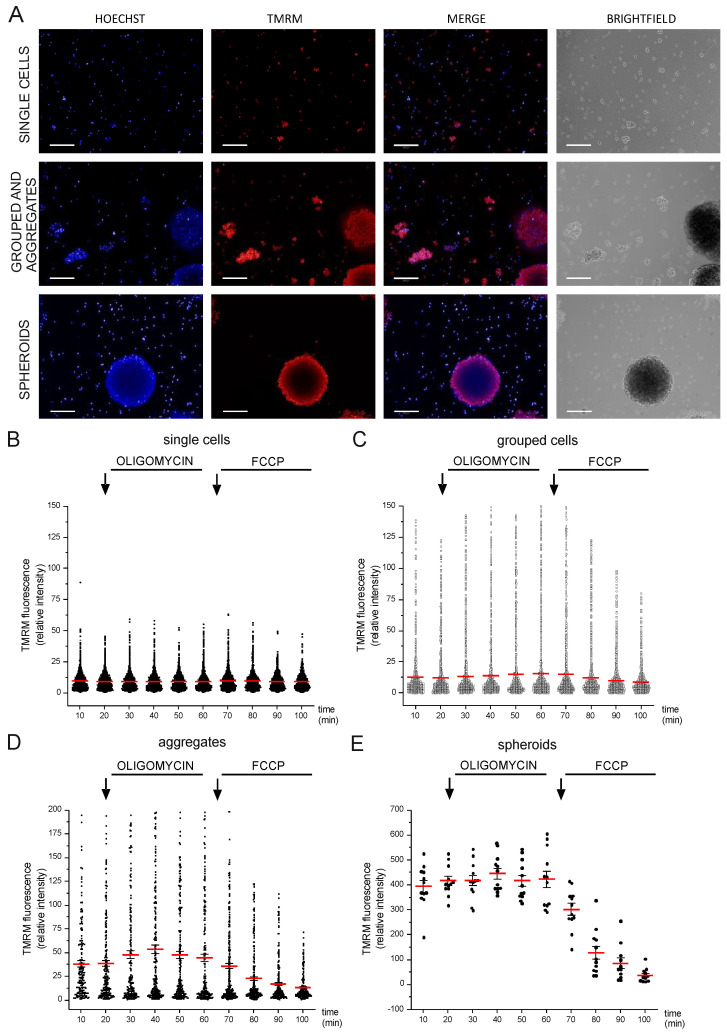
High-content imaging of mitochondrial membrane potential in 3D cell models. (**A**) Representative images of TMRM, Hoechst, and brightfield of LUHMES cells; scale bar: 200 μm. TMRM fluorescence intensity analysis of different classes: (**B**) single cells, (**C**) grouped, (**D**) aggregates, and (**E**) spheroids. The values reported in the graph have been normalized on basal levels. Data represent the mean ± SEM of 3 biological replicates.

We measured ΔΨm at all the different timepoints: interestingly, we found that in basal conditions, ΔΨm was different depending on the aggregation state of the cells. Their response to oligomycin and FCCP also varied within the different groups (Figure 2 and Figure S3B). Specifically, in single cells, mitochondria were not either hyperpolarized or depolarized upon oligomycin and FCCP exposure; however, cells became more responsive alongside the increase in their aggregation state. Changes in the TMRM kinetic were more evident when cells were clustered as spheroids.

### 3.3. ΔΨm Analysis in Isolated Muscle Fibers

**Figure 3:**
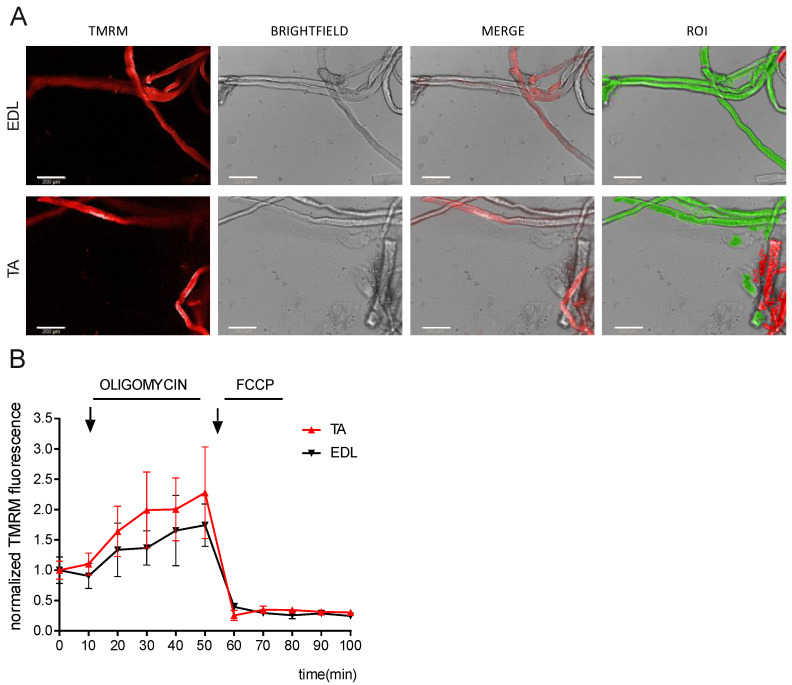
High-content microscopy of ΔΨm kinetics in isolated muscle fibers. (**A**) Fluorescent microscopy images of TMRM of muscle fibers isolated from extensor digitorum longus (EDL; **top**) and tibialis anterior (TA; **bottom**) muscles. Intact, unblemished myofibers appear as translucent cylinders with striated patterns (as shown in the brightfield images). White scale bars: 200 μm. (**B**) Mitochondrial membrane potential (TMRM fluorescence intensity) was quantified and analyzed in the selected individual myofibers. Values are normalized to basal intensity levels. Data represent the mean ± SEM of 3 biological replicates.

Another complex 3D model to be analyzed via high-content microscopy is an isolated tissue. We set up an imaging and analysis workflow to detect ΔΨm in freshly isolated muscle fibers from the extensor digitorum longus (EDL) and tibialis anterior (TA) muscles composed mostly of fast-glycolytic myofibers (Figure 3A). ΔΨm both in basal conditions and after exposure to oligomycin and FCCP was similar in both fiber subtypes. Compared to basal conditions, 5 μM oligomycin led to hyperpolarization, while uncoupling mitochondria with 10 μM FCCP-induced depolarization, as shown by the decrease in the average TMRM fluorescence intensity (Figure 3B). Three-dimensional reconstruction of Z-stacks coupled to phenoLOGIC^TM^ machine learning analysis (Perkin Elmer analysis algorithm) was used to detect each individual fiber (Supplementary Figure S4).

### 3.4. Development of Machine Learning Approach to Distinguish Cell Populations in Co-Cultures

Subsequently, we verified whether the developed high-content microscopy and analysis methods were also suitable for co-cultures of cell populations with similar/overlapping morphological features. We co-cultured the melanoma cell line A375 with human monocyte-derived macrophages, which were obtained from healthy donors. Before analyzing TMRM kinetics, we set up a method to correctly distinguish the two cell populations in an unbiased approach and in the absence of cell surface markers.

Cell-type-specific markers are not always available; their expression levels may be variable within the same population due to the physiological status of the cells (e.g., activated versus resting macrophages etc.) or may influence cell behavior. To overcome this issue, we designed an approach independent of cell-specific markers. We hypothesized that both morphology and texture properties of nuclei staining could differ based on the cell type. Therefore, we stained both cell types with Hoechst and TMRM and performed image segmentation analysis (Supplementary Figure S5). Then, by means of principal component analysis (PCA) and machine learning algorithms, we identified the key parameters necessary to separate the two cell populations (macrophages from cancer cells) upon staining with Hoechst. Then, we used the machine learning algorithm Deep Neural Network (DNN) built by repeating a pair of Dropout and Dense layers five times (Supplementary Datasets S1 and S2). For simplicity, the same model was used for all the combinations between population A (A375 cells) and B (macrophage from a single donor), as shown in Supplementary Dataset S3. DNN networks were implemented by using the Keras framework [21] with TensorFlow back-end [22] and optimized both in the choice of the hyper-parameters and in the number and type of used features. For both cell lines, the performance appeared slightly lower while still being able to correctly classify more than 95% of the cells (data not shown). For the final analysis, we took into account all the available features, as described above (the corresponding Python scripts are available as Supplementary Dataset S4). With this approach, we were able to assign to each cell a score (ranging from 0 to 1), which indicates the probability of belonging to a specific cell population (e.g., either cancer cells or macrophages). In this scenario, the higher the score assigned to individual cells, the higher the probability of belonging to the target population. Then, to avoid any potential artifact, we filtered the results based on the score assigned to each individual cell and then discarded all the objects classified as “macrophages” or “cancer cells” with a score lower than 0.6 from our statistical analyses.

### 3.5. High-Content Imaging of ΔΨm in Co-Cultures: A Machine Learning Approach

**Figure 4:**
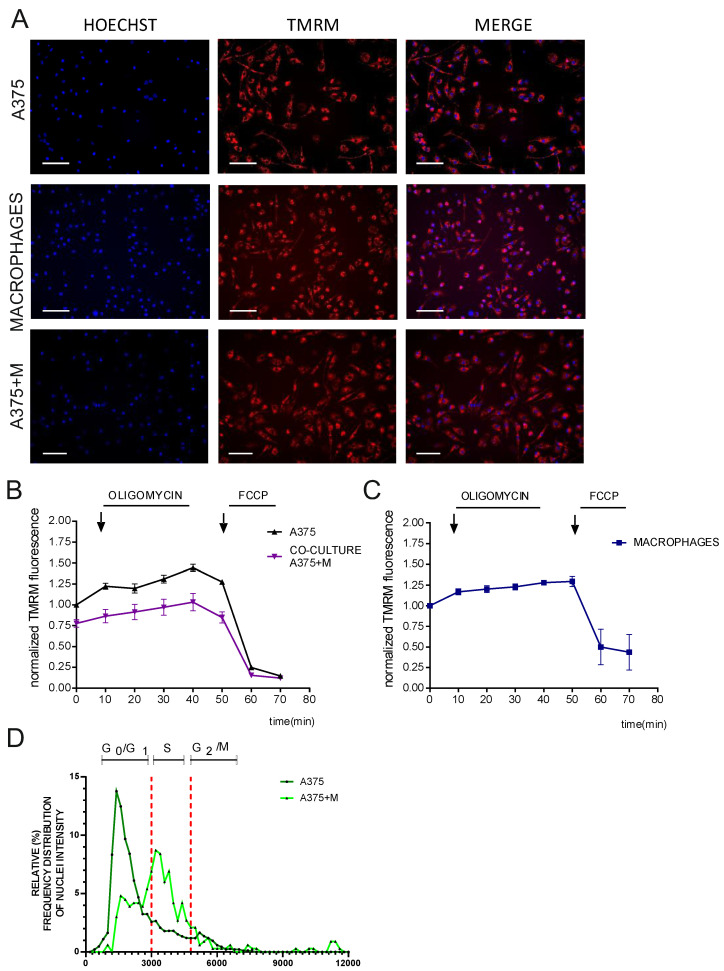
High-content imaging of ΔΨm in cell co-cultures and machine learning quantification. (**A**) Representative images of melanoma A375 cells (cultured either alone or co-cultured) and macrophages treated with TMRM. White scale bars: 200 μm. (**B**,**C**) the respective TMRM analysis in basal conditions or exposed to oligomycin and FCCP. Data represent the mean ± SEM from 4 biological replicates. (**D**) Cell cycle phase distribution of melanoma A375 cells cultured either alone or in co-culture with macrophages. Dashed lines indicate the division of the three cell cycle phases: G0/G1, S, and G2/M. Representative graph reports the percentage of the frequency distribution of nuclei intensity in each cell cycle phase. Data represent the mean ± SEM from 4 biological replicates.

After establishing the method for cell classification, we performed an unbiased evaluation of membrane potential at the single-cell level in response to oligomycin and FCCP via high-content imaging (Figure 4A). We analyzed the kinetics of cell populations both in monoculture and in co-culture (Figure 4B,C). To compare the datasets, we normalized TMRM intensity values of A375 (in either mono- or co-culture) to the basal TMRM intensity value of A375 alone. Interestingly, we observed that the ΔΨm of the melanoma cells differed in the presence of macrophages (Figure 4B). Specifically, A375 melanoma cells co-cultured with macrophages exhibited a decreased response to oligomycin compared to melanoma cells culture. We observed a similar trend also with a non-cancer cell line (HEK T293, data not shown).

Finally, we took advantage of the Hoechst staining to provide further information on the changes in the physiology of melanoma A375 cells in the presence of macrophages. We observed that the cancer cells shifted their cell cycle progression to the S phase when in co-culture; whereas when they were cultured alone, they were mostly in the G2/M phase (Figure 4D). This shift indicates a change in A375 cell proliferation rates when they interact with macrophages.

## 4. Discussion

ΔΨm is often considered to be a proxy of mitochondria activity/well-being. So far, kinetic measurements of ΔΨm have been set up at the single-cell level in standard fluorescent microscopes in vivo by means of positron emission tomography [24] and super-resolution microscopy [13,14]. However, analysis of ΔΨm kinetics in live cells by means of high-throughput microscopy has not been optimized yet. To fill this methodological gap, we took advantage of high-content imaging coupled to automated image analysis and machine learning approaches. We developed a reproducible and unbiased high-content microscopy methodology to study cell populations that is suitable for several different cell types and conditions, both in 2D and 3D models. Our method, which considers phenotyping analysis combined with machine learning, is applicable to many 2D models, such as immortalized cell lines, primary cell cultures, single cells, and cell clusters, as well as 3D models such as spheroids and isolated muscle fibers, and finally, co-cultures of different cell models.

Firstly, we set up a high-content analysis to monitor ΔΨm kinetics in single-cell monolayers and verified the effect of oligomycin and FCCP as positive controls. We then optimized the measurement for 3D models by analyzing the kinetics of ΔΨm in spheroids and muscle fibers. Surprisingly, in neural precursor cells LUHMES, we observed an increased TMRM signal according to the aggregation state of the cells. This could be explained by considering their stemness and differentiation potential, as shown for other cells. For example, human cardiac mesenchymal progenitor cells with high in vitro differentiation capacity are characterized by high ΔΨm [25]. Furthermore, hematopoietic stem cells with high self-renewal capacity are characterized by low ΔΨm [26].

Another factor to consider is that different cell aggregation states affect the partial oxygen pressure and distribution, thereby influencing mitochondrial respiration [27,28]. Indeed, it has been shown that 3D cell aggregates are characterized by regions of hypoxia or anoxia that are exacerbated by the size of the cell cluster, media height, and oxygen consumption rates. Thus, the ΔΨm differences that we observed in Luhmes-derived 3D models could also be due to inconstant oxygen pressure.

Simultaneous staining of the cells with different dyes makes it possible to monitor and correlate various subcellular parameters in the same sample. As proof of concept, we used melanoma A375 cells grown either alone or with macrophages with Hoechst and TMRM and analyzed both ΔΨm and the cell cycle phase. To distinguish the two cell populations, we took advantage of several Hoechst parameters (such as intensity, morphology, texture, symmetry, and threshold properties of nuclei; Supplementary Dataset S3). By using Principal Component Analysis (PCA) and machine learning, we were able to distinguish and classify both cell types. We found that ΔΨm kinetics and the cell cycle progression of melanoma A375 cells differed when cultured alone or co-cultured with macrophages. A similar result was also observed in the non-cancer cell line HEK (data not shown). We also observed that macrophages can alter the cell cycle progression of melanoma cells from G0/G1 to S phase arrest, highlighting the possibility of performing multiple analyses within a single experiment. However, *why* and *how precisely* this change occurs is beyond the scope of this work.

To summarize, we demonstrated that we were able to measure both the ΔΨm and the progression of the cell cycle of melanoma cells in the presence of macrophages, opening the possibility for new strategies for the characterization of immune cell–cancer cell interaction in vitro, eventually based also on other classifier and/or cellular parameters.

## Data Availability

The data that support the findings of this study are available from the corresponding author, M.G., upon reasonable request.

## Supplementary Materials

The following supporting information can be downloaded at: https://www.mdpi.com/article/10.3390/cells12071089/s1, Figure S1: Workflow of the analysis protocol of high-content microscopy images of human dermal and lung fibroblasts; Figure S2, Workflow of analysis protocol in high-content microscopy of LUHMES cells and 3D spheroids; Figure S3, Representative images of 3D Spheroids in all the channels and statistical analysis of the 4 different groups of cells; Figure S4, Workflow of analysis protocol in high-content microscopy of 3D myofibers; Figure S5; Workflow of analysis protocol in high-content microscopy of co-cultured melanoma A375 cells and macrophages; Supplementary Dataset S1, DNN_Classification.py: the major script, necessary for training and generation of the DNN; Supplementary Dataset S2, Utilities.py: a set of functions to complement the main script; Supplementary Dataset S3, confM1.py: this dataset contains an example of data to configure the network; Supplementary Dataset S4, DNN_Eval.py: a script to generate output evaluations.
