# Supplementary material for: High-Throughput Microscopy Analysis of Mitochondrial Membrane Potential in 2D and 3D Models
Source: Cells. 2023 Apr 5;12(7):1089. doi: 10.3390/cells12071089 (PMC10093082; doi:10.3390/cells12071089)
Supplement: Supplementary file 1 [file cells-12-01089-s001.zip › cells-2295913-supplementary.pdf]

cells

MDPI

Supplementary Figure S1

Step	Input	Method	Output
Find Nuclei	**Channel :** HOECHST 33342<br>**ROI :** None	**Method :** C<br>Common Threshold : 0.2<br>Area : > 30 μm^2<br>Splitting Coefficient : 1<br>Individual Threshold : 0.32<br>Contrast : > 0.32	Output Population : Nuclei
Calculate Morphology Properties	**Population :** Nuclei<br>**Region :** Nucleus	**Method :** Standard<br>Area<br>Roundness	Property Prefix : Nucleus
Calculate Intensity Properties	**Channel :** HOECHST 33342<br>**Population :** Nuclei<br>**Region :** Nucleus	**Method :** Standard<br>Mean	Property Prefix : Intensity Nucleus HOECHST 33342
Select Population	**Population :** Nuclei	**Method :** Filter by Property<br>Nucleus Roundness : > 0.7	Output Population : Nuclei Selected
Find Cytoplasm	**Channel :** TMRM<br>**Nuclei :** Nuclei Selected	**Method :** A<br>Individual Threshold : 0.08	
Select Cell Region	**Population :** Nuclei Selected	**Method :** Resize Region [%]<br>Region Type : Ring Region<br>Outer Border : -45 %<br>Inner Border : -35 %	Output Region : BG
Calculate Intensity Properties (2)	**Channel :** TMRM<br>**Population :** Nuclei Selected<br>**Region :** Cytoplasm	**Method :** Standard<br>Mean	Property Prefix : Intensity TMRM
Calculate Intensity Properties (3)	**Channel :** TMRM<br>**Population :** Nuclei Selected<br>**Region :** BG	**Method :** Standard<br>Mean	Property Prefix : Intensity BG TMRM
Calculate Properties	**Population :** Nuclei Selected	**Method :** By Formula<br>Formula : A-B<br>Variable A : Intensity TMRM Mean<br>Variable B : Intensity BG TMRM Mean	Output Property : Delta TMRM
Select Population (2)	**Population :** Nuclei Selected	**Method :** Filter by Property<br>Delta TMRM : > 0	Output Population : TMRM positive

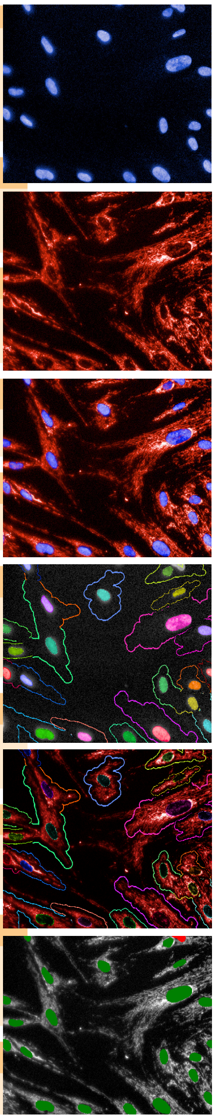

Supplementary Figure S1. Workflow of the analysis protocol of high-content microscopy images of human dermal and lung fibroblasts**.** TMRM and Hoechst fluorescence images of human fibroblasts (HDFa, IMR-90) were acquired using a 40× objective with the automated high-content fluorescence imaging Operetta® system (PerkinElmer; HiTS@UniPD facility). Scale bar, 50 µm. Image analysis was performed in a stepwise manner: nuclei detection was followed by automated identification of the cell area; a ring region surrounding the cell boundaries was used as background for each object. Single object as well as average per well fluorescence intensity was calculated for each timepoint.

Supplementary Figure S2

A

Find Image Region	**Channel :** HOECHST 33342<br>**ROI :** None	**Method :** Common Threshold<br>Threshold : <u>0.3</u><br>Split into Objects<br>Area : > <u>2</u> px²	Output Population : Cell Clusters<br>Output Region : Cluster ROI
Calculate Morphology Properties	**Population :** Cell Clusters<br>**Region :** Cluster ROI	**Method :** Standard<br>Area<br>Roundness<br>Width<br>Length<br>Ratio Width to Length	Output Properties : Cluster ROI
Find Nuclei	**Channel :** HOECHST 33342<br>**ROI :** Cell Clusters<br>**ROI Region :** Cluster ROI	**Method :** M<br>Diameter : <u>12</u> µm<br>Splitting Coefficient : <u>0.35</u><br>Common Threshold : <u>0.09</u>	Output Population : Nuclei
Find Cytoplasm (2)	**Channel :** TMRM<br>**Nuclei :** Nuclei	**Method :** A<br>Individual Threshold : <u>0.05</u><br>Restrictive Region : Cluster ROI	
Select Region	**Population :** Nuclei<br>**Region :** Cytoplasm	**Method :** Resize Region [%]<br>Outer Border : <u>-60</u> %<br>Inner Border : <u>-20</u> %	Output Region : BG
Calculate Morphology Properties (4)	**Population :** Nuclei<br>**Region :** Nucleus	**Method :** Standard<br>Area<br>Roundness<br>Width<br>Length<br>Ratio Width to Length	Output Properties : Nucleus
Select Population (2)	**Population :** Cell Clusters	**Method :** Filter by Property<br>Cluster ROI Area [µm²] : <= <u>150</u>	Output Population : Single Cell
Calculate Intensity Properties (3)	**Channel :** HOECHST 33342<br>**Population :** Single Cell<br>**Region :** Nucleus	**Method :** Standard<br>Mean<br>Contrast	Output Properties : Intensity single cells HOECHST 33342
Calculate Intensity Properties (7)	**Channel :** TMRM<br>**Population :** Single Cell<br>**Region :** Cytoplasm	**Method :** Standard<br>Mean	Output Properties : Single cell TMRM intensity
Select Region (2)	**Population :** Single Cell<br>**Region :** Cell	**Method :** Resize Region [%]<br>Outer Border : <u>-80</u> %<br>Inner Border : <u>-40</u> %	Output Region : Bg single cell
Calculate Intensity Properties	**Channel :** TMRM<br>**Population :** Single Cell<br>**Region :** Bg single cell	**Method :** Standard<br>Mean	Output Properties : Intensity Bg single cell TMRM
Calculate Properties	**Population :** Single Cell	**Method :** By Formula<br>Formula : A-B<br>Variable A : Single cell TMRM intensity Mean<br>Variable B : Intensity Bg single cell TMRM Mean	Output Property : Delta TMRM Single Cell
Select Population (3)	**Population :** Cell Clusters	**Method :** Filter by Property<br>Cluster ROI Area [µm²] : > <u>150</u><br>Cluster ROI Area [µm²] : < <u>1100</u><br>Boolean Operations : F1 and F2	Output Population : Grouped Cells
Calculate Intensity Properties (5)	**Channel :** HOECHST 33342<br>**Population :** Grouped Cells<br>**Region :** Nucleus	**Method :** Standard<br>Mean	Output Properties : Intensity Nucleus Grouped HOECHST 33342
Find Spots	**Channel :** HOECHST 33342<br>**ROI :** Grouped Cells	**Method :** D<br>Detection Sensitivity : <u>1</u><br>Splitting Coefficient : <u>0.9</u><br>Background Correction : <u>0.7</u><br>Calculate Spot Properties	Output Population : Nuclei Spot Grouped Cells

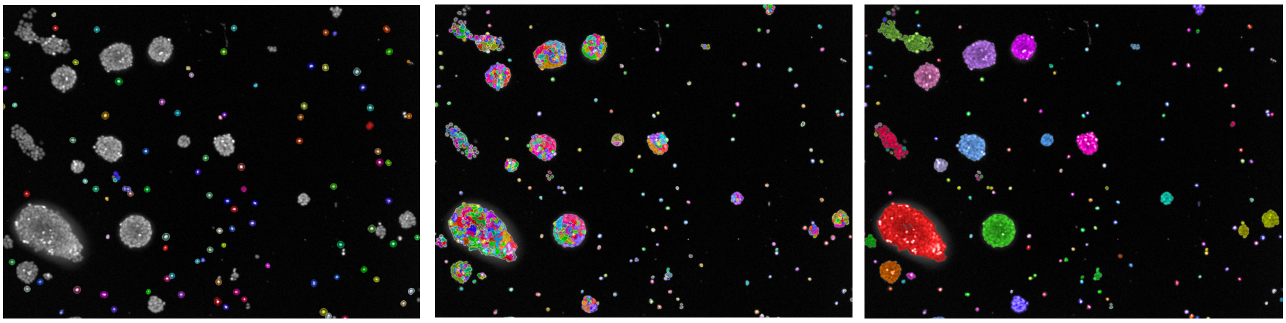

B

Calculate Intensity Properties (13)	**Channel :** HOECHST 33342<br>**Population :** Grouped Cells<br>**Region :** Spots	**Method :** Standard<br>Mean	Output Properties : HOECHST Nuclei spot Grouped
Calculate Intensity Properties (8)	**Channel :** TMRM<br>**Population :** Grouped Cells<br>**Region :** Cytoplasm	**Method :** Standard<br>Mean	Output Properties : Intensity Cytoplasm Grouped TMRM
Select Region (3)	**Population :** Grouped Cells<br>**Region :** Cell	**Method :** Resize Region [%]<br>Outer Border : -80 %<br>Inner Border : -40 %	Output Region : Grouped Cells Bg
Calculate Intensity Properties (2)	**Channel :** TMRM<br>**Population :** Grouped Cells<br>**Region :** Grouped Cells Bg	**Method :** Standard<br>Mean	Output Properties : Intensity Grouped Cells Bg TMRM
Calculate Properties (2)	**Population :** Grouped Cells	**Method :** By Formula<br>Formula : A-B<br>Variable A : Intensity Cytoplasm Grouped TMRM Mean<br>Variable B : Intensity Grouped Cells Bg TMRM Mean	Output Property : Delta TMRM grouped cell
Select Population (4)	**Population :** Cell Clusters	**Method :** Filter by Property<br>Cluster ROI Area [µm²] : >= 1100<br>Cluster ROI Roundness : > 0.8<br>Boolean Operations : F1 and F2	Output Population : spheroids
Select Population	**Population :** Cell Clusters	**Method :** Filter by Property<br>Cluster ROI Area [µm²] : > 1100<br>Cluster ROI Roundness : <= 0.8<br>Boolean Operations : F1 and F2	Output Population : Big Spheroids
Calculate Intensity Properties (10)	**Channel :** HOECHST 33342<br>**Population :** Big Spheroids<br>**Region :** Nucleus	**Method :** Standard<br>Mean	Output Properties : Intensity Big spheroids HOECHST 33342
Calculate Intensity Properties (11)	**Channel :** TMRM<br>**Population :** Big Spheroids<br>**Region :** Cytoplasm	**Method :** Standard<br>Mean	Output Properties : Intensity Cytoplasm spheroidsTMRM
Calculate Morphology Properties (3)	**Population :** Big Spheroids<br>**Region :** Cluster ROI	**Method :** Standard<br>Area<br>Roundness<br>Width<br>Length<br>Ratio Width to Length	Output Properties : Big Spheroids
Select Region (5)	**Population :** Big Spheroids<br>**Region :** Cell	**Method :** Resize Region [%]<br>Outer Border : -80 %<br>Inner Border : -40 %	Output Region : Big Spheroids Bg
Calculate Intensity Properties (12)	**Channel :** TMRM<br>**Population :** Big Spheroids<br>**Region :** Big Spheroids Bg	**Method :** Standard<br>Mean	Output Properties : Intensity Big Spheroids Bg TMRM
Calculate Properties (4)	**Population :** Big Spheroids	**Method :** By Formula<br>Formula : A-B<br>Variable A : Intensity Cytoplasm spheroidsTMRM Mean<br>Variable B : Intensity Big Spheroids Bg TMRM Mean	Output Property : Delta TMRM Big Spheroids
Find Spots (3)	**Channel :** HOECHST 33342<br>**ROI :** Big Spheroids	**Method :** D<br>Detection Sensitivity : 1<br>Splitting Coefficient : 0.9<br>Background Correction : 0.089<br>Calculate Spot Properties	Output Population : Nuclei spot Big spheroids
Calculate Intensity Properties (14)	**Channel :** HOECHST 33342<br>**Population :** Big Spheroids<br>**Region :** Spots	**Method :** Standard<br>Mean	Output Properties : HOECHST Nuclei spot Big Spheroids
Calculate Intensity Properties (6)	**Channel :** HOECHST 33342<br>**Population :** spheroids<br>**Region :** Nucleus	**Method :** Standard<br>Mean<br>Contrast	Output Properties : Intensity spheroids HOECHST 33342
Calculate Intensity Properties (9)	**Channel :** TMRM<br>**Population :** spheroids<br>**Region :** Cytoplasm	**Method :** Standard<br>Mean	Output Properties : Intensity TMRM spheroids
Find Spots (2)	**Channel :** HOECHST 33342<br>**ROI :** spheroids	**Method :** D<br>Detection Sensitivity : 1<br>Splitting Coefficient : 0.9<br>Background Correction : 0.7<br>Calculate Spot Properties	Output Population : Nuclei spot Spheroids
Calculate Intensity Properties (15)	**Channel :** HOECHST 33342<br>**Population :** spheroids<br>**Region :** Spots	**Method :** Standard<br>Mean	Output Properties : HOECHST Nuclei spots Spheroids
Calculate Intensity Properties (4)	**Channel :** TMRM<br>**Population :** spheroids<br>**Region :** Spheroids Bg	**Method :** Standard<br>Mean	Output Properties : Intensity Spheroids Bg TMRM
Calculate Properties (3)	**Population :** spheroids	**Method :** By Formula<br>Formula : A-B<br>Variable A : Intensity TMRM spheroids Mean<br>Variable B : Intensity Spheroids Bg TMRM Mean	Output Property : Delta TMRM Spheroids
Calculate Morphology Properties (2)	**Population :** Big Spheroids<br>**Region :** Cluster ROI	**Method :** Standard<br>Area<br>Roundness<br>Width<br>Length<br>Ratio Width to Length	Output Properties : Big Spheroids

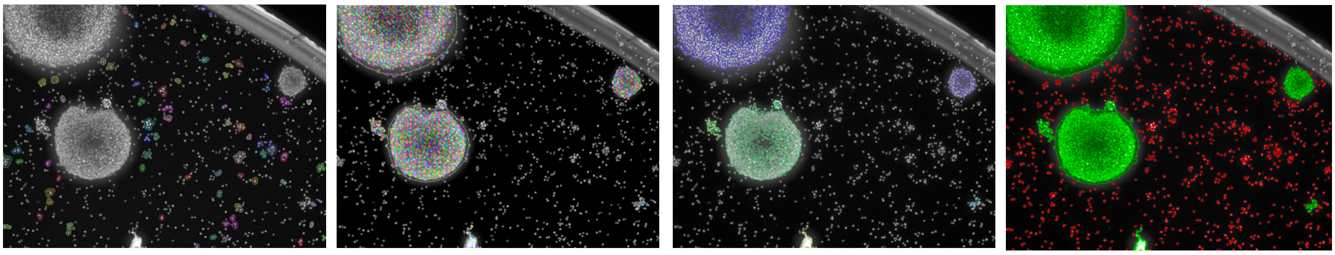

Supplementary Figure S2. Workflow of analysis protocol in high-content microscopy of LUHMES cells and 3D spheroids. Imaging of mitochondrial membrane potential and nuclei in LUHMES cells was performed with the automated platform (Operetta® High-Content Imaging System, PerkinElmer; HiTS@UniPD facility). A) Hoechst staining was used for image segmentation and detection of ROIs. B) The Hoechst-positive ROIs of the LUHMES cells were categorized into 4 groups: single cells,

grouped, big aggregates and spheroids, as shown in the right column. The classes were distinguished based on the size of the nuclei clusters, and TMRM intensity was calculated per each class.

Supplementary Figure S3

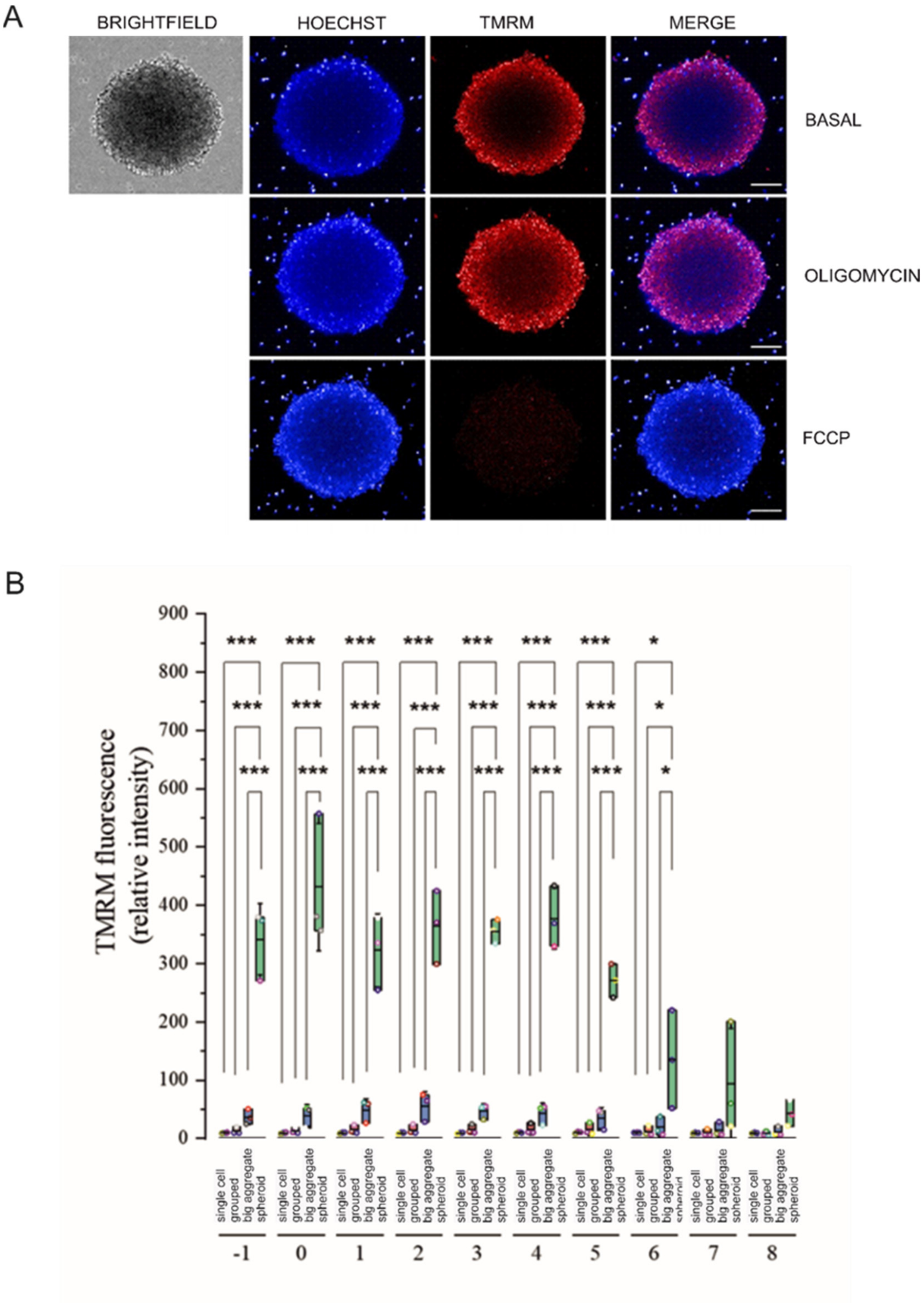

Supplementary Figure S3. Representative images of 3D Spheroids in all the channels and statistical analysis of the 4 different groups of cells. (A) Representative images of mitochondria and nuclei staining in LUHMES 3D Spheroids at three different timepoints, corresponding to basal conditions, exposure to oligomycin and FCCP. (B) Box-plot graph of TMRM fluorescence intensity values (arbitrary units) representing of ΔΨm changes occurring in the four populations at each time-point. Data represent the mean ± SEM of 3 independent experiments. *$p < 0.05$, ***$p < 0.001$. Standard ANOVA procedures followed by multiple pairwise comparison adjusted with Bonferroni corrections were performed using GraphPad software (GraphPad Software, San Diego, CA, USA).

Supplementary Figure S4

	Input	Method	Output
Filter Image	**Channel :** Brightfield	**Method :** Sliding Parabola<br>Curvature : 3	Output Image : Sliding Parabola Brightfield Fiber
Find Image Region	**Channel :** Sliding Parabola Brightfield Fiber<br>**ROI :** None	**Method :** Local Threshold<br>Threshold : 0.45<br>Region Scale : 6 µm<br>Closing : 0.2 µm<br>Filling : Fill Plane-Wise<br>Smoothing : 4 µm<br>Volume : > 4000000 µm³	Output Population : ROIs<br>Output Region : ROIs region
Calculate Morphology Properties (3)	**Population :** ROIs<br>**Region :** ROIs region	**Method :** Standard<br>Volume<br>Surface Area<br>Number of Fragments<br>Equivalent Ellipsoid Axes<br>Object Box Size<br>Sphericity<br>Inner Sphere Radius<br>Object Height<br>Maximum Thickness<br>Footprint Area<br>Maximum Crosssection Area<br>Maximum Inner Disk Radius	Property Prefix : ROIs region
Calculate Texture Properties (4)	**Channel :** Sliding Parabola Brightfield Fiber<br>**Population :** ROIs<br>**Region :** ROIs region	**Method :** PLS Features<br>Filter : Plane Bright<br>Scale XY : 2 px<br>Scale Z : 0 px<br>PSF Aspect Ratio : 3<br>Kernel Normalization	Property Prefix : ROIs region Sliding Parabola Brightf<br>Fiber
Find Image Region (2)	**Channel :** TMRM<br>**ROI :** ROIs<br>**ROI Region :** ROIs region	**Method :** Absolute Threshold<br>Lowest Intensity : ≥ 100<br>Highest Intensity : ≤ INF<br>Closing : 6 µm<br>Filling : Fill Cavities<br>Smoothing : 12 µm<br>Volume : > 300000 µm³	Output Population : Fibers<br>Output Region : Fibers Region
Calculate Intensity Properties	**Channel :** TMRM<br>**Population :** Fibers<br>**Region :** Fibers Region	**Method :** Standard<br>Mean<br>Standard Deviation<br>Coefficient of Variance<br>Median	Property Prefix : Intensity Fibers Region TMRM
Select Region	**Population :** Fibers<br>**Region :** Fibers Region	**Method :** Resize Region [µm/px]<br>Direction : XYZ<br>Fixed Aspect Ratio : 1<br>Outer Border XY : -180 µm<br>Outer Border Z : -180 µm<br>Inner Border XY : -150 µm<br>Inner Border Z : -150 µm	Output Region : Fibers Resized
Calculate Intensity Properties (2)	**Channel :** TMRM<br>**Population :** Fibers<br>**Region :** Fibers Resized	**Method :** Standard<br>Mean<br>Standard Deviation	Property Prefix : Intensity Bg TMRM
Calculate Properties	**Population :** Fibers	**Method :** By Formula<br>Formula : A-B<br>Variable A : Intensity Fibers Region TMRM Mean<br>Variable B : Intensity Bg TMRM Mean	Output Property : Delta TMRM
Calculate Texture Properties	**Channel :** Brightfield<br>**Population :** Fibers<br>**Region :** Fibers Region	**Method :** PLS Features<br>Filter : All<br>Scale XY : 1 px<br>Scale Z : 0 px<br>PSF Aspect Ratio : 2<br>Kernel Normalization	Property Prefix : Fibers Region Brightfield
Select Population (5)	**Population :** Fibers	**Method :** Filter by Property<br>Delta TMRM : > 10<br>Intensity Fibers Region TMRM Mean : > 10<br>Intensity Fibers Region TMRM CV [%] : > 20<br>Boolean Operations : F2 and F3 and F4	Output Population : True Fibers

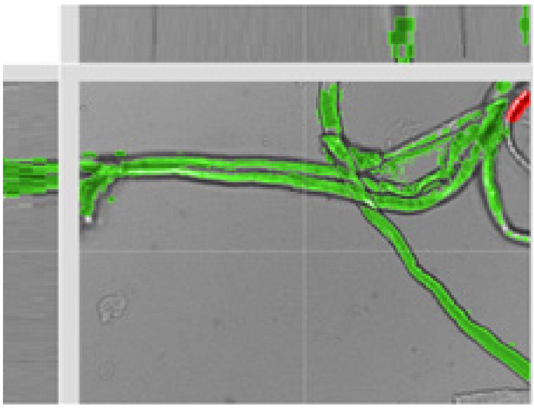

Supplementary Figure S4. Workflow of analysis protocol in high-content microscopy of 3D myofibers. Imaging of mitochondrial membrane potential in isolated muscle fibers from EDL and TA were acquired by automated fluorescence microscopy. White scale bars: 200 µm. Image analysis was performed in a stepwise manner. Brightfield image correction was followed by 3D reconstruction. Texture analysis of the latter allowed the first segmentation, that is detection of image regions containing fibers. These areas have been further segmented based on phenoLOGIC™ analysis (Perkin-Elmer machine learning algorithm), to identify individual fibers. Contracted fibers (likely damaged due to the dissociation process) were discarded based on morphology and texture parameters, yielding the final population named “true fibers".

Supplementary Figure S5

A

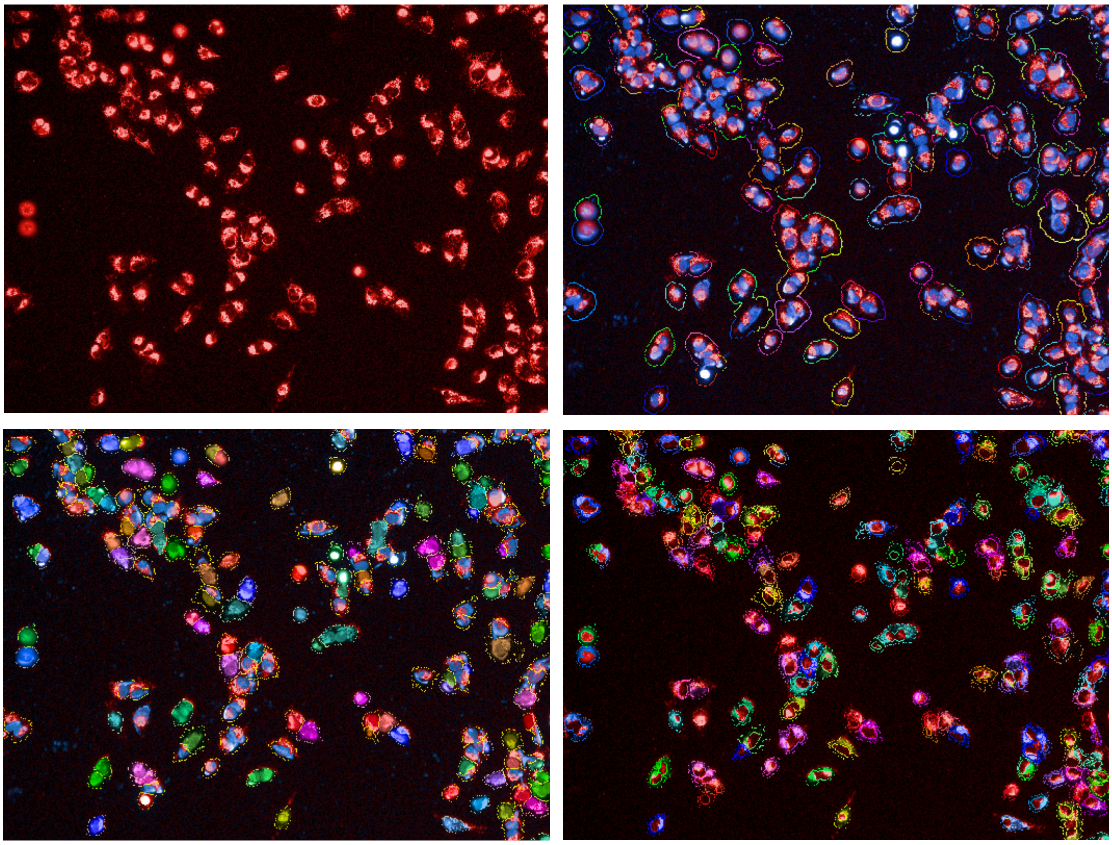

B

	Input	Method	Output
Find Nuclei	**Channel :** HOECHST 33342<br>**ROI :** None	**Method :** M<br>Diameter : 20 µm<br>Splitting Sensitivity : 0.46<br>Common Threshold : 0.11	Output Population : Nuclei
Find Cytoplasm	**Channel :** TMRM<br>**Nuclei :** Nuclei	**Method :** B<br>Common Threshold : 0.38<br>Individual Threshold : 0.33	
Select Cell Region	**Population :** Nuclei	**Method :** Resize Region [%]<br>Region Type : Cytoplasm Region<br>Outer Border : -25 %<br>Inner Border : -5 %	Output Region : Background
Calculate Intensity Properties	**Channel :** TMRM<br>**Population :** Nuclei<br>**Region :** Cytoplasm	**Method :** Standard<br>Mean	Property Prefix : TMRM Int Cyt
Calculate Intensity Properties (3)	**Channel :** TMRM<br>**Population :** Nuclei<br>**Region :** Background	**Method :** Standard<br>Mean	Property Prefix : Intensity Background TMRM
Calculate Properties	**Population :** Nuclei	**Method :** By Formula<br>Formula : A-B<br>Variable A : TMRM Int Cyt Mean<br>Variable B : Intensity Background TMRM Mean	Output Property : Delta TMRM
Calculate Morphology Properties	**Population :** Nuclei<br>**Region :** Cell	**Method :** Standard<br>Area<br>Roundness<br>Width<br>Length<br>Ratio Width to Length	Property Prefix : Cell
Calculate Texture Properties (2)	**Channel :** TMRM<br>**Population :** Nuclei<br>**Region :** Cytoplasm	**Method :** SER Features<br>Scale : 0 px<br>Normalization by : Kernel<br>SER Spot<br>SER Hole<br>SER Edge<br>SER Ridge<br>SER Valley<br>SER Saddle<br>SER Bright<br>SER Dark	Property Prefix : Cytoplasm TMRM
Calculate Texture Properties	**Channel :** HOECHST 33342<br>**Population :** Nuclei<br>**Region :** Nucleus	**Method :** SER Features<br>Scale : 0 px<br>Normalization by : Kernel<br>SER Spot<br>SER Hole<br>SER Edge<br>SER Ridge<br>SER Valley<br>SER Saddle<br>SER Bright<br>SER Dark	Property Prefix : Nucleus HOECHST 33342
Calculate Intensity Properties (2)	**Channel :** HOECHST 33342<br>**Population :** Nuclei<br>**Region :** Cell	**Method :** Standard<br>Mean<br>Standard Deviation<br>Coefficient of Variance	Property Prefix : HOECHST Intensity
Calculate Morphology Properties (2)	**Population :** Nuclei<br>**Region :** Nucleus	**Method :** Standard<br>Area<br>Roundness<br>Width<br>Length<br>Ratio Width to Length	Property Prefix : Nucleus

Supplementary Figure S5. Workflow of analysis protocol in high-content microscopy of co-cultured melanoma A375 cells and macrophages. A) TMRM and Hoechst fluorescence images of human melanoma A375 cells and macrophages, grown either alone or in co-culture, were acquired by automated fluorescence microscope platform (Operetta® High Content Imaging System, PerkinElmer; HiTS@UniPD facility). Scale bar 100 µm. B) Hoechst staining was used for image segmentation; several nuclei parameters (texture, intensity, morphology) were measured and considered for the subsequent machine learning analysis.
